# Nanofiber-Supported Palladium Nanocubes—Toward Highly Active and Reusable Catalyst

**DOI:** 10.1021/acsomega.3c08414

**Published:** 2024-01-11

**Authors:** Justyna Kalisz, Kamil Sobczak, Krzysztof Maksymiuk, Agata Michalska, Jan Krajczewski

**Affiliations:** † Faculty of Chemistry, University of Warsaw, Pasteura 1, 02-093 Warsaw, Poland; ‡ Biological and Chemical Research Centre, University of Warsaw, Żwirki i Wigury 101, 02-089 Warsaw, Poland

## Abstract

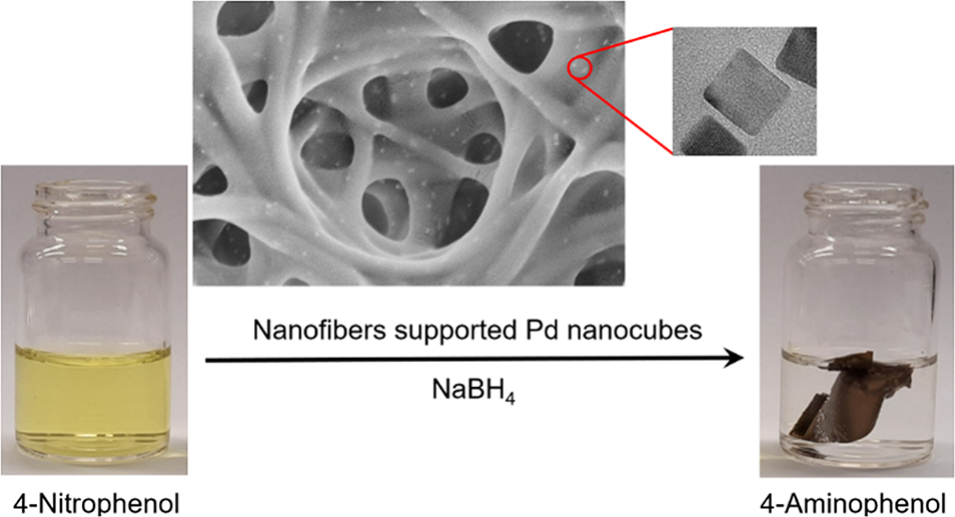

Electrospun nanofibers were used to support palladium nanocubes, resulting in a highly active, stable, and reusable catalyst. The system proposed herein offers significant advantages compared to catalysts in the form of nanoparticles suspension. The porous, solvent permeable structure of the nanofiber mat ensures uniform and stable time distribution of palladium nanoparticles; preventing coalescence and allowing multiple use of the catalyst. The proposed cross-linked poly(vinyl alcohol) nanofiber mat loaded with Pd nanocubes during the nanofiber preparation step is a macroscopic structure of intrinsically nanostructural character of the catalyst that can be easily transferred between different solutions without compromising its effectiveness in consecutive cycles. Thus, obtained system was characterized with high catalytic activity as tested on a model example of 4-nitrophenol (4-NP) reduction by NaBH_4_ to 4-aminophenol (4-AP). It is shown that loading nanofibers with Pd nanocubes during electrospinning resulted in a significantly more stable system compared to surface modification of obtained nanofibers with nanocube suspension.

## 2 Introduction

Nanostructural metals are reaching significant attention as highly active heterogeneous catalysts.^1^ This is observed due to significantly higher surface-to-volume ratio that surpasses that of their bulk counterparts.^1^ For example, nanoparticles of platinum,^2^ palladium,^3−7^ silver,^8−10^ gold,^11,12^ or their alloys^3^ (with Ni,^13^ Co,^14^ Cu^15^) show high catalytic activity for model compound 4-nitrophenol (4-NP) conversion. It should be stressed that for catalytic purposes, the shape of nanoparticles is important, e.g., nanocubes of Pd show improved performance compared to other geometries.^16−19^ However, typically, the drawback of nanoparticles suspension type catalysts is the tendency to be deactivated by the presence of agents (e.g., metal–organic frameworks, zeolites, and organic micelles) used to mitigate the coalescence.^1^ Moreover, suspension of catalytically active nanoparticles is in some respect more similar to a quasihomogeneous catalyst than to a heterogeneous catalyst, making reuse of nanoparticles relatively difficult. Toward this end, different strategies have been proposed to immobilize nanoparticles with ultimate goal to obtain material of similarly high catalytic activity.

Surprisingly, the immobilization of catalytically active nanoparticles within polymeric nanofibers structure was not broadly considered.^20,21^ This approach in principle offers significant advantages: it allows obtaining uniform distribution of nanoparticles within the porous structure and at the same time preventing coalescence and exposing maximum of the active surface to enhance catalytic activity. On the other hand, nanofiber mats, despite the nanostructural character, are a macroscopic structure of total volume limited only by the amount of material applied. In principle, a catalyst of this format can be easily transferred from one solution to another, contributing to better use of resources such as metals nanoparticles. Nanoparticles embedded within the nanofibers offer a surface free from stabilizing agents that can contribute to higher catalytic activity.

In general, two approaches can be used to incorporate metal nanoparticles in the nanofiber mat: nanoparticles (or other additives, e.g., enzymes) can be added to the polymer suspension to be electrospun, resulting in the bulk presence of the metal particulates in the nanofibers and consecutively in the mat. This approach potentially can result also in higher stability of the whole system,^20^ which ultimately leads to nanofibers offering unique catalytic properties.^22^ A variation of this approach that can be used to prepare metal nanoparticles containing nanofiber mats is electrospinning of a polymer with a precursor of, e.g., metal nanoparticles, followed by another step (or steps) required to prepare metal particulates within the structure.^23^ The disadvantage of this approach is, however, poor control of geometry of metal structures formed and in some cases (e.g., when metal ions are introduced post nanofibers mat formation) their distribution within the mat.

An alternative approach is to modify obtained nanofibers with nanoparticles (applied from suspension);^20,24−26^ however, also in this case, the control of distribution of metal nanoparticles within the mat is limited. Clearly, to have control on the geometry of structures included in the mat as well as to ensure uniform distribution within the nanofiber bulk, using polymer suspension with ready structures is the safer option. Last but not the least, the possibility to reuse the catalyst without significant loss in its activity is highly important, especially if nanoparticles supported on other materials are considered.

The choice of polymer applied to prepare the nanofibers seems vitally important. Often, nanofiber mats intended for catalysis purposes and containing metal nanoparticles embedded in the nanofiber formation processes were calcinated to obtain catalysts.^27^ If the polymer applied is intended to support the catalyst during the reaction performed, e.g., in the aqueous phase, the nanofiber mat needs to be well wettable, which unfortunately is not always easy for some polymers in the form of nanofibers. Thus, the rational choice can be application of hydrophilic polymers such as, e.g., poly(vinyl alcohol), which enhances stability and allows reuse of the structure in solution that can be cross-linked.

To characterize obtained catalysts, including nanofibers, the conversion of 4-NP is often used as a model system. This approach is justified by toxicity of the 4-NP pollutant commonly found in water.^28,29^ Advantageously, 4-NP can be catalytically degraded in the presence of NaBH_4_ to form 4-aminophenol.^30^

For example, catalysis on, e.g., silver^31^ or platinum nanoparticles^32^ (deposited by atomic layer deposition, ALD) on polyacrylonitrile fiber or fibers modified with palladium nanoparticles (Pd NPs), by electrospinning, has been proposed in several cases and was carried out after the electrospinning of the polymer mat.^33,34^

The aim of the present work is to propose a new and effective catalytic material based on supporting highly efficient and well-defined Pd nanocubes within the electrospun poly(vinyl alcohol) (PVA) nanonofibers, cross-linked after preparation. The rationale for using Pd nanostructures is that this catalyst is less susceptible to poisoning effects compared to, e.g., platinum, which is currently only 20% less expensive; thus, Pd offers a good effect to cost ratio. Last but not the least, using noble metal imbedded in the nanofiber mat not only makes it reusable as catalyst but also allows easier recycling of palladium used. The catalytic activity and stability of the proposed material was tested on a model of 4-nitrophenol reduction to 4-aminophenol.

## 3 Experimental Section

### 3.1 Materials

PVP (40 kDa), Mowiol (PVA, Mw 130 kDa), sodium borohydride, sodium tetrachloropalladate (II) (Na_2_PdCl_4_), ascorbic acid (AA, < 99%), 4-nitrophenol, and potassium bromide were purchased from Sigma-Aldrich. Citric acid was obtained from Chempur. All materials were of high purity and were used as received without further purification or treatment. Water was purified by a Millipore Milli-Q system and had a resistivity of 18 MΩ cm.

### 3.2 Pd Nanocube Formation

Pd nanocubes were formed by a wet-chemical method described earlier and slightly modified.^35−37^ For this purpose, 37 mg of PVP, 50 mg of AA and 400 mg of KBr were dissolved in 8 mL of deionized water and heated to 80 °C for 20 min under magnetic stirring. In another flask, 57 mg of Na_2_PdCl_4_ was dissolved in 3 mL of deionized water. Subsequently, both solutions were mixed and kept at 80 °C for 4 h under magnetic stirring. The nanoparticles were then purified by centrifugation at 8000 rpm for 10 min and refilled with water. The weight content of Pd NPs in 1 mL of suspension was ca. 6.4 mg (nanoparticles were weighed after evaporation of water)

### 3.3 Nanofiber Formation

Nanofiber mats containing Pd NPs were prepared using an electrospinning method. First, a suspension of PVA (180 mg) with citric acid (18 mg) was dissolved in 1.5 mL of water using a magnetic stirrer heated to 60 °C for 24 h. Separately, 2 mL of Pd NPs was centrifuged (18000 rpm) and dispersed in 0.5 mL of water using an ultrasonic homogenizer probe. Then, the polymer suspension was mixed with Pd NPs and left for 30 min on a magnetic stirrer.

The electrospinning apparatus consisted of a DC power source (ELSR30P300,Technix). The high voltage was connected to a stainless steel needle (27 G) attached to a plastic syringe with a polymeric suspension. The flow rate of the polymeric suspension was 0.65 mL/h, and its value was controlled by a syringe pump (KDS100, *K*_d_ Scientific). Fiber mats were collected on an electrically grounded target of aluminum foil. The voltage applied during electrospinning nanofibers mat preparation was set at 27 kV, with the distance between the needle and the collector equal to 15 cm. Electrospinning was carried out at 21 °C with humidity of 55% controlled by a dehydrator and air conditioning. The fiber mats were collected for 3 h during the electrospinning process. After electrospinning, the nanofiber mats (c.a. 205 mg and 140 cm^2^) were left in a laboratory atmosphere overnight. Next day, PVA nanofiber mats were cross-linked for 1 h in a furnace (160 °C). The weight content of Pd NPs in nanofiber mats calculated from the contents of all slurry components used for electrospinning (except the solvent that evaporates) was about 6%. Nanofiber mats without Pd NPs were prepared in the same condition except for the voltage during electrospinning, which was 14 kV. PVA-PdNPs film was prepared by drop-casting of the polymer suspension of the same composition as for electrospinning onto aluminum foil, dried overnight, and cross-linked for 1 h in a furnace (160 °C).

The amount of Pd NPs in nanofibers was optimized (results not shown); the highest available contents from the point of view of nanofiber formation by electrospinning was used.

### 3.4 Nitrophenol Decomposition

The catalytic properties of Pd nanocubes were examined by analysis of the kinetics of 4-nitrophenol reduction by sodium borohydride. Typically, 3 mL of water and 20 μL of 0.01 M aqueous solution of 4-nitrophenol and Pd catalyst were placed into a 10 mm PMMA cuvette (with nominal volume of 3.5 mL) from Starna Cells, Inc. In the next step, 300 μL of 0.1 M freshly prepared solution of sodium borohydride were added. The final concentrations of 4-NP and NaBH_4_ in the cuvette were 60 μM and 9 mM, respectively. After this, the color of the solution suddenly changed from pale yellow to tight yellow, due to the formation of 4-nitrophenolate ions. To the cuvette, 2.2 mg of PVA-PdNPs fiber mat or 2.2 mg of PVA-Pd NPs film or 21 μL of Pd NPs was added (equivalent weight amount of Pd catalyst). The catalytic decomposition of 4-NP to 4-AP was conducted in a cuvette or vial (with a total capacity of vial 26 mL, the volumes and weight of used substrates increased 4 times compared to the experiments performed in the cuvettes). To investigate the decomposition of nitroaromatic compounds, UV–vis absorption spectra were recorded in the spectral range between 250 and 500 nm in time.

### 3.5 Characterization Method

Scanning electron microscopy (SEM) analyses of the nanoparticles formed were carried out by using a Merlin (Zeiss, Germany) field emission scanning electron microscope (SEM) equipped with an energy-dispersive X-ray spectroscopy probe (Bruker). Samples of electrospun fibers (both as prepared ones and after contact with aqueous phase, followed by drying for 24 h in lab atmosphere) were coated with Au/Pd using an Emitech sputter coater for 15 s.

UV–vis extinction spectra were recorded by using a Shimadzu UV-2401PC spectrophotometer. The spectra were collected in the range 500–250 nm, with the spectral gap equal to 1 nm, with the data interval equal to 1 nm.

TEM investigations were conducted using a Talos F200X transmission microscope at 200 kV. The measurements were performed in STEM mode using a high-angle annular dark-field (HAADF) detector. The energy-dispersive X-ray spectroscopy (EDX) was used for chemical composition mapping. The specimens for TEM investigations were suspended in ethanol/water and dropped onto amorphous thin carbon embedded on a Cu grid. The nanofibers before TEM imaging were electrospun on Cu grid (thin carbon) by 2 s. Then nanofibers on a Cu grid were dried overnight and cross-linked for 1 h in a furnace (160 °C).

The water contact angle measurements were carried out with a digital microscope (Delta Optical Smart 5MP PR) using 2 μL of water drop. The measurement of the contact angle was repeated 6 times; the obtained angle values were averaged; and SD was calculated.

## 4 Results and Discussion

### 4.1 Pd Nanocube Characterization

**Figure 1:**
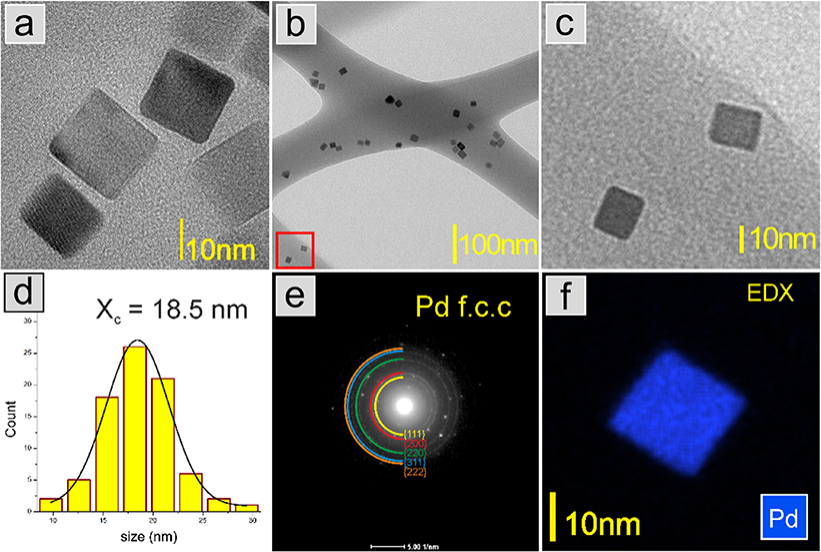
TEM images of cube shaped Pd NPs (a), nanofiber mats with Pd NPs after cross-linking, enlargement of the part of the image (b) marked with a red frame (c), histogram of the size of particle (d), electron diffraction pattern together with the identification of the crystal structure of Pd f.c.c (e), and EDX map with chemical distribution of palladium (f).

Pd cubic nanostructures have been prepared. TEM studies confirmed the presence of well-formed cube-shaped palladium particles as shown in Figure 1a. It was also tested whether the effect of temperature (160 °C) during PVA cross-linking affects the shape of nanoparticles in the fiber. The images shown in Figure 1b,c clearly confirm that the shape of the Pd nanoparticles is not affected by the temperature treatment after cross-linking, Pd nanocubes are clearly seen within the PVA nanofibers. Additionally, it can be seen that the nanoparticles are uniformly distributed in the nanofiber structure. Based on the TEM images, a histogram of the size of the nanoparticles was made, which is presented in Figure 1d. By fitting the histogram to the Gaussian distribution, an average nanoparticle size of 18.5 nm was obtained. Based on the electron diffraction method, the existence of one crystalline phase assigned to the typical Pd f.c.c. structure was revealed (Figure 1e). The EDX studies presented in Figure 1f confirm the presence of Pd without impurities.

### 4.2 Nanofiber Characterization

The PVA polymer suspension containing Pd NPs used for electrospinning was intensively dark. The obtained nanofiber mat with Pd NPs was slightly brown (Figure S1a); after cross-linking, it became darker (Figure S1b).

**Figure 2:**
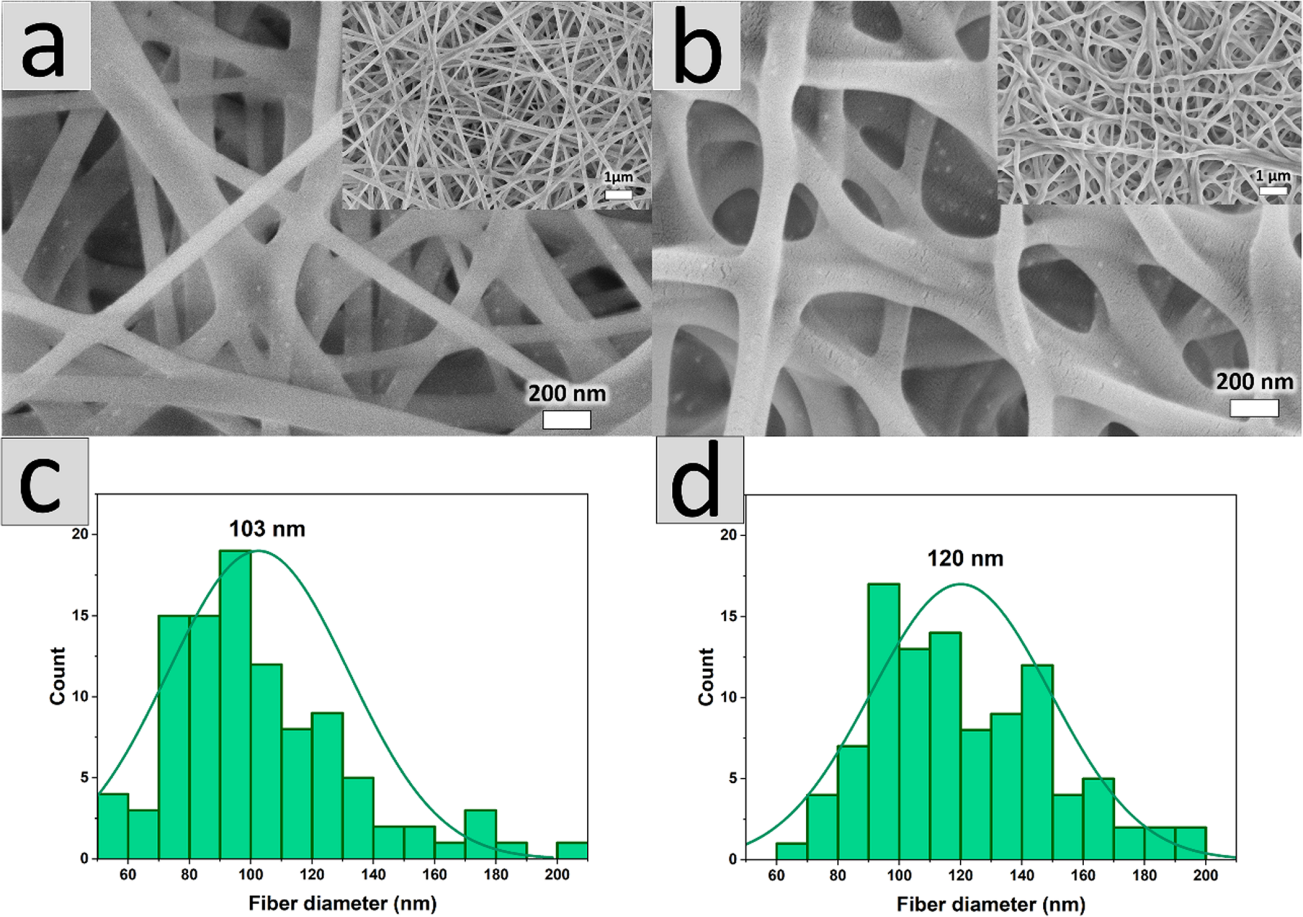
SEM images of nanofiber mat with Pd NPs after cross-linking (a) before and (b) after contact with water; (c, d) histograms presenting average size of fibers shown above in pictures (a) and (b), respectively.

To enhance the stability of prepared nanofiber mats and allow reusability of the catalyst in the aqueous phase, PVA was cross-linked. Citric acid (added to polymeric suspension before electrospinning) was applied as a cross-linker in a heat driven process (1 h, 160 °C). The cross-linked mats were found to be stable in water (non-cross-linked mats dissolved instantaneously in contact with water). SEM images of cross-linking of PVA show Pd NP nanocubes (Figure 2) as brighter spots. The mat was uniform, with the dominant diameter of nanofibers slightly higher than 100 nm, Figure 2c. Nanofibers dried in the laboratory atmosphere after contact with water were slightly twisted, yet the nanostructural character of the mat was well preserved (Figure 2b). The mean diameter of nanofibers was slightly increased to ca. 120 nm, Figure 2d. This confirms the successful cross-linking of the PVA in the nanostructure. It should be stressed that Pd nanocubes are clearly seen in Figure 2b, proving that nanostructures are well retained in the nanofiber mat despite the contact with the aqueous phase.

The EDX analysis confirmed the presence of C, O, and Pd in the cross-linked nanofiber mats, Figure S2. The w/w ratio of Pd in cross-linked PVA nanofibers is c.a. 8.8%, i.e., it is well comparable with value calculated from the mass of the individual components used to prepare mat (6%).

In order to test the obtained catalytic material, Pd PVA fibers with citric acid after cross-linking will be sufficiently permeable to aqueous solutions; the water contact angle for this material was measured. The obtained contact angle value of 40° ± 1 indicates the high hydrophilicity of the obtained mat and proves that it will be easily permeable to aqueous solutions.

### 4.3 Catalytic Activity toward 4-NP Decomposition

**Figure 3:**
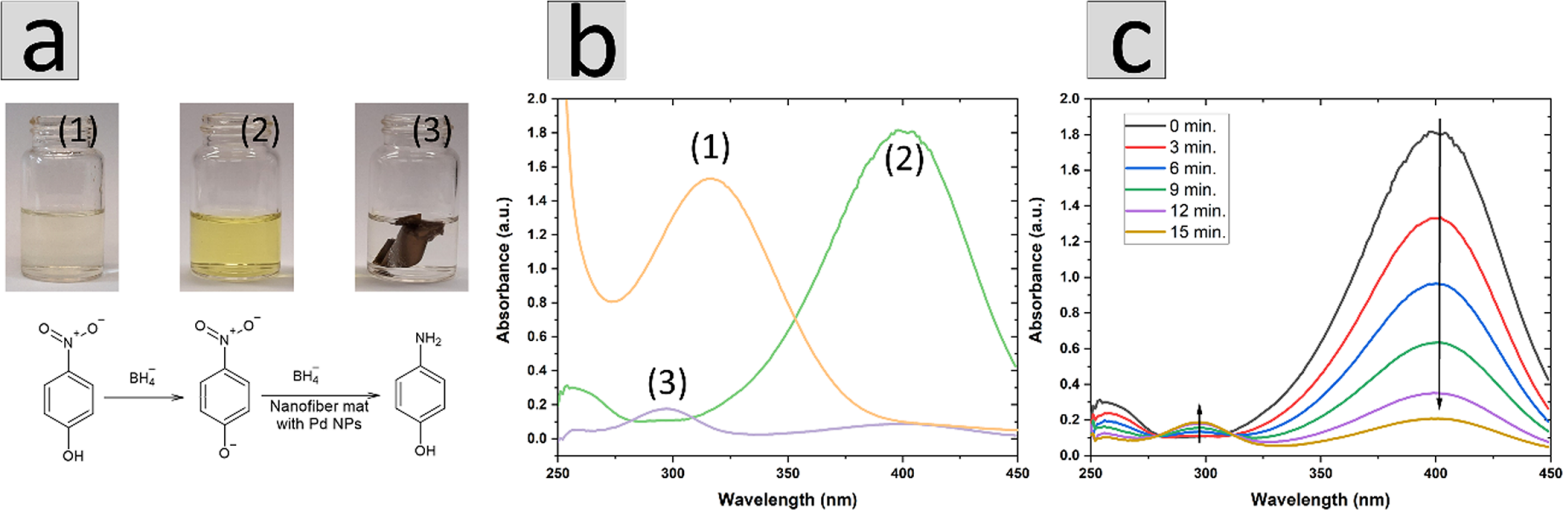
(a) Photo of fresh solution of 4-NP (1), after addition of sodium borohydride (in absence of catalyst) (2), 12 min after introduction of nanofiber mat with Pd NPs (3), and scheme of reduction reaction of 4-NP to 4-AP. (b) Spectra UV–vis of 4-NP (orange line), 4-NP with NaBH_4_ (2), and 4-AP (3) (c) Typical UV–vis spectra recorded during 4-NP decomposition in the presence of nanofiber mat with Pd NPs (1st cycle).

The Pd NPs supported on nanofibers were used in the model process of catalysis of 4-NP decomposition with colorimetric detection of the reaction progress. The aqueous solution of 60 μM 4-nitrophenol is pale yellow (Figure 3a-1): the UV–vis absorption spectrum of 4-nitrophenol is dominated by the broad band with maximum located at 316 nm (orange line in Figure 3b-1). Addition of sodium borohydride to the solution turns color to intense bright yellow (Figure 3a-2), which is caused by the formation of the phenolic salt with maximum absorption at 399 nm (green line in Figure 3b-2). The mixture of solutions of 4-nitrophenol and sodium borohydride does not change color for many hours, and the recorded UV–vis spectra do not show any significant change even after 3 days (results not shown). However, in the presence of Pd nanocubes, in the nanofibers solution containing 4-nitrophenol and sodium borohydride, the bright yellow color quickly fades (Figure 3a-3) as a result of the reduction of 4-nitrophenolate ions to 4-aminophenol with the strong absorption band at 303 nm (Figure 3b-3).^30^

The catalytic decomposition of 4-NP to 4-AP was conducted in a cuvette or vial; right after introducing the nanofiber mat with Pd NPs into the cuvette, hydrogen was intensively produced on the catalyst surface. This process was clearly seen as bubble formation in the whole volume of the cuvette. The color of the solution quickly faded, and the obtained pale color was retained for a longer period of time (in this particular system, the solution color did not fade completely). One can assume that intensive hydrogen production resulting from sodium borohydride decomposition on the catalyst surface (i.e., bubbles of hydrogen seen by the bare eye, which can be removed by mixing) is blocking the active place of the catalyst for the reaction. Figure S3a shows the absorbance measurement without stirring of solution.

For this purpose, the reaction mixture was intensively stirred by magnetic stirrer with 1500 rpm for the whole reaction time. Under the condition of stirring visually, the observed initial color quickly fades, and the complete discoloration of the solution occurs. The overall change of the color from intense yellow to colorless was observed in the time of 15 min in first cycle (Figure 3a-3). The scheme of the reaction taking place with the PVA-PdNPs mat is presented in Figure 3a.

The reaction of Pd NPs supported on nanofibers was monitored, recording UV–vis spectra every 3 min. As time elapses, a decrease in peak intensity at 400 nm was observed as well as formation of a new peak located at 303 nm, corresponding to the formation of 4-AP (Figure 3c).

In a control experiment, activity of Pd nanocubes supported on nanofibers was compared with catalytic activity of nanoparticles suspension. The analysis of UV–vis spectra showed that catalytic activity of Pd NPs in suspension is similar to that of the same amount of Pd NPs immobilized in the polymer nanofiber (comparable to reaction rate for the fourth and fifth cycles recorded for the mat, Figure S3b,c). Thus, it was confirmed that the immobilization of Pd NPs within the nanofiber does not limit the catalytic activity.

The catalytic activity is obviously a very important parameter for catalysts; however, the possibility to reuse the catalyst without a decrease in its activity, i.e., high chemical stability of the catalyst in time is extremely important, too. The proper selection of polymer applied to trap Pd nanoparticles, e.g., using cross-linked PVA, allows contact of the nanocatalyst with the aqueous phase and due to applied cross-linking, prevents dissolution of catalyst, ultimately allowing reuse of the system.

**Figure 4:**
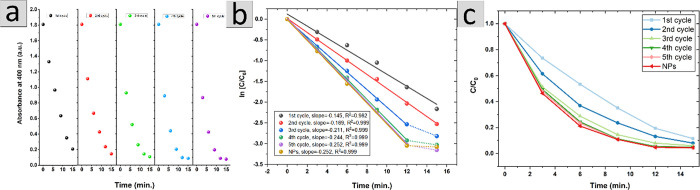
(a) Temporal evolution of the absorption at 400 nm for nanofiber mat with Pd NPs in the following 4-NP decomposition cycle in the function of time, (b) relationship between ln(*c*(*t*)/*c*(0)) and reaction time, where *A*(*t*) and *A*(0) are the absorbance of the reaction mixture at time *t* and at time 0, measured at 400 nm; (c) relation between concentration to *c*(0) ratio and reaction time.

The proposed nanofiber mat with Pd NPs in principle allows for the reusability of the catalyst in many cycles. The dependences of absorbance at 400 nm on the duration of the catalytic process were tested during 5 cycles, and the results are shown in Figure 4a. Similar shapes of dependence of peak height (proportional to 4-NP concentration) on time for all cycles were recorded, pointing to the high stability of the proposed catalyst. The 4-NP in first cycle is almost completely reduced after 15 min, while starting from the third cycle, this time is significantly shorter, close to 12 min, Figure 4. However, for the fourth and fifth cycles, the rate of 4-NP decomposition is similar, slightly faster than for the initial cycles. These results show that the nanofibers supported nanoparticles do not lose their activity allowing reuse and preserving almost 100% catalytic activity. Additionally, it is easy to transfer the catalyst in the form of a mat to a fresh solution of 4-NP with NaBH_4_.

Assuming that the reduction of 4-NP was performed in the presence of NaBH_4_ excess, the reaction rate is independent of its concentration and could be considered as a pseudo-first order with respect to 4-NP. Therefore, it would be possible to determine the apparent rate constant *k*_app_ [min^–1^] from the slope of linear dependence ln[*C*/*C*_0_] on the time. The obtained dependences, Figure 4b, are linear, confirming the assumption of the pseudo-first reaction order; the values of *k*_app_ determined from the slopes of these dependences are 0.145, 0.169. 0.211, 0.244, 0.252, and 0.252 [min^–1^] for the first to fifth cycles and Pd NPs, respectively. Basing on the determined *k*_app_ for the fourth and fifth cycles, the half-life time is 2.75 min, denoting that after 20 min over 99% of 4-NP undergoes reduction. However, for the first cycle, some deviations from linearity can be seen in Figure 4b, presenting that dependences of the reactant concentration (related to initial concentration, Figure 4c) on time show an almost linear dependence for the first cycle for shorter reaction time (below 8 min.). This effect suggests that for this cycle and in this time range, the reaction rate is almost independent of 4-NP concentration. It can be assumed that this effect is most likely due to poor wetting of the nanofiber mat structure and thus the limited access of the reactant to the catalyst, with the rate limiting step (rds). For longer times, when the mat is fully equilibrated with water, the rds changes to that described by a typical first-order kinetic equation.

Although values of rate constant for Pt and Pd nanostructural catalyst were reported,^6^ in the case of nanoparticles supported on the surface of nanofibers, a decrease in kinetics was observed over time, which could have resulted from the detachment of Au^31^ or Pt^32^ nanoparticles from the fiber surface; in both cases, the nanoparticles were added to the fiber mats after the electrospinning process. Therefore, trapping in the interior of the fibers ensures stable catalysis in many cycles, without a decrease in its kinetics. On the other hand, slightly higher rate constants were recorded earlier,^23,34^ which are results of higher concentration of NaBH_4_ and/or accumulation of nanoparticles mainly on the polymer surface in the mentioned papers.

**Figure 5:**
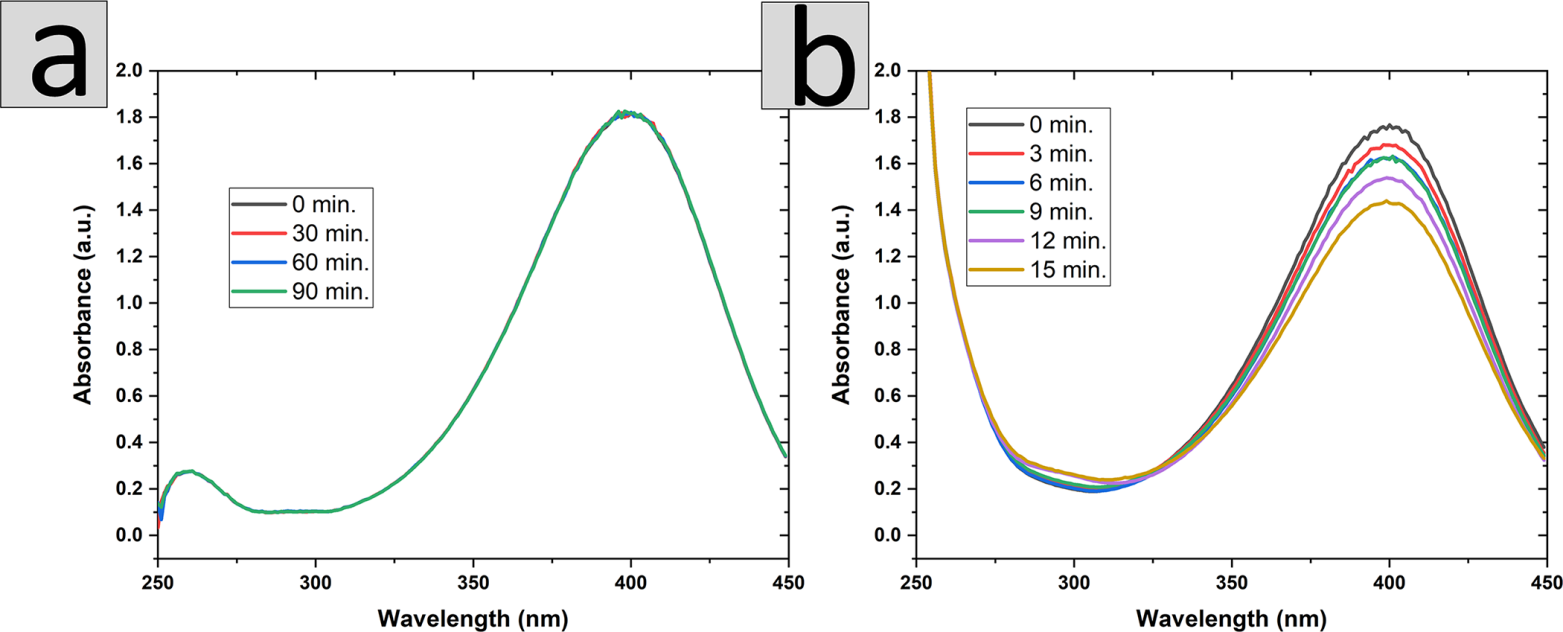
UV–vis spectra showing the change of 4-NP concentration in (a) nanofiber mat without Pd NPs and (b) polymer film with Pd NPs.

In a control experiment, the catalytic behavior of a PVA cross-linked nanofiber mat prepared without Pd NPs was tested. The UV–vis spectra of the reaction mixture were recorded after 30 min. No color changes were observed even until 90 min. This indicates that in the absence of Pd nanostructures the process of reduction is not observed (Figure 5a). In addition, the impact of large surface development with nanofiber mats on the kinetics of 4-NP degradation was comparable to that of the PVA-PdNPs film. The progress of the reaction in the case of a relatively flat film is slower than for the nanofiber mat after 15 min less than 25% of the 4-NP was converted to 4-AP (Figure 5b).

## 5 Conclusions

A unique highly active and stable catalyst based on Pd nanocubes supported on a PVA nanofiber mat is proposed. The studies on the model system of 4-NP reduction to 4-AP by NaBH_4_ clearly confirm high catalytic activity, retained up to 5 cycles, clearly proving that this system is reusable. It has been shown that the supporting nanoparticles on the PVA cross-linked nanofiber mat do not adversely affect the catalytic properties of palladium nanocubes; moreover, this system provides appropriate conditions for effective catalysis. The proposed system of macroscopic size retains intrinsic properties of the nanostructural catalyst distributed within the bulk of the nanofibers mat.

## Supporting Information Available

Photos of the obtained nanofiber mat with Pd NPs before and after cross-linking, EDS spectra of PVA-PdNPs fiber mat, and UV–vis spectra recorded during 4-NP decomposition in the presence of nanofiber mat or with Pd NPs (PDF)
