# Supplementary material for: Nanofiber-Supported Palladium Nanocubes—Toward Highly Active and Reusable Catalyst
Source: ACS Omega. 2024 Jan 11;9(3):4050–6. doi: 10.1021/acsomega.3c08414 (PMC10809285; doi:10.1021/acsomega.3c08414)
Supplement: Supplementary file 1 — ao3c08414_si_001.pdf [file ao3c08414_si_001.pdf]

Supporting Information

Nanofibers supported palladium nanocubes - towards highly active and reusable catalyst

Justyna Kalisz[a*], Kamil Sobczak[b], Krzysztof Maksymiuk[a], Agata Michalska[a], Jan Krajczewski[a*]

a Faculty of Chemistry, University of Warsaw

b Biological and Chemical Research Centre**,** University of Warsaw

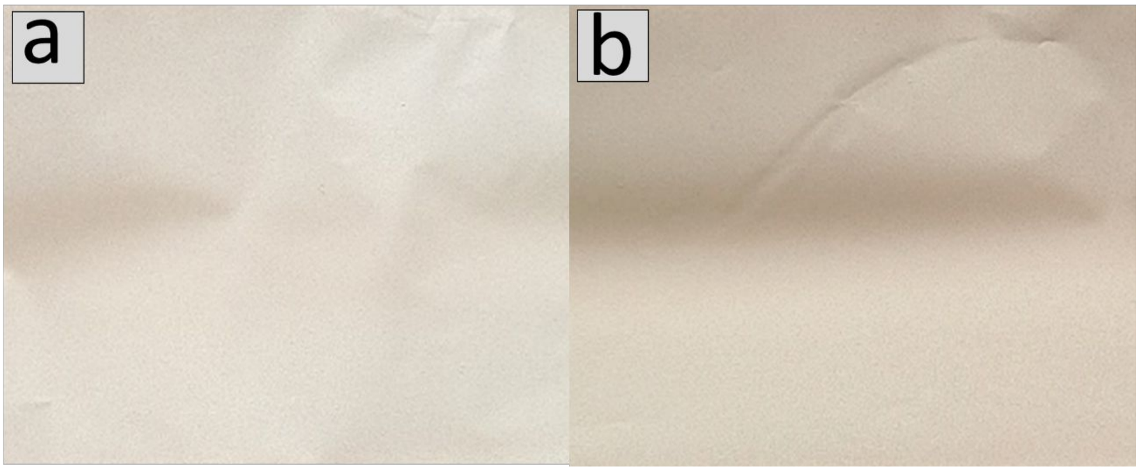

Figure. S1 The photos of obtained nanofiber mat with Pd NPs before (a) and after cross-linking (b). (photos were taken by J. Kalisz).

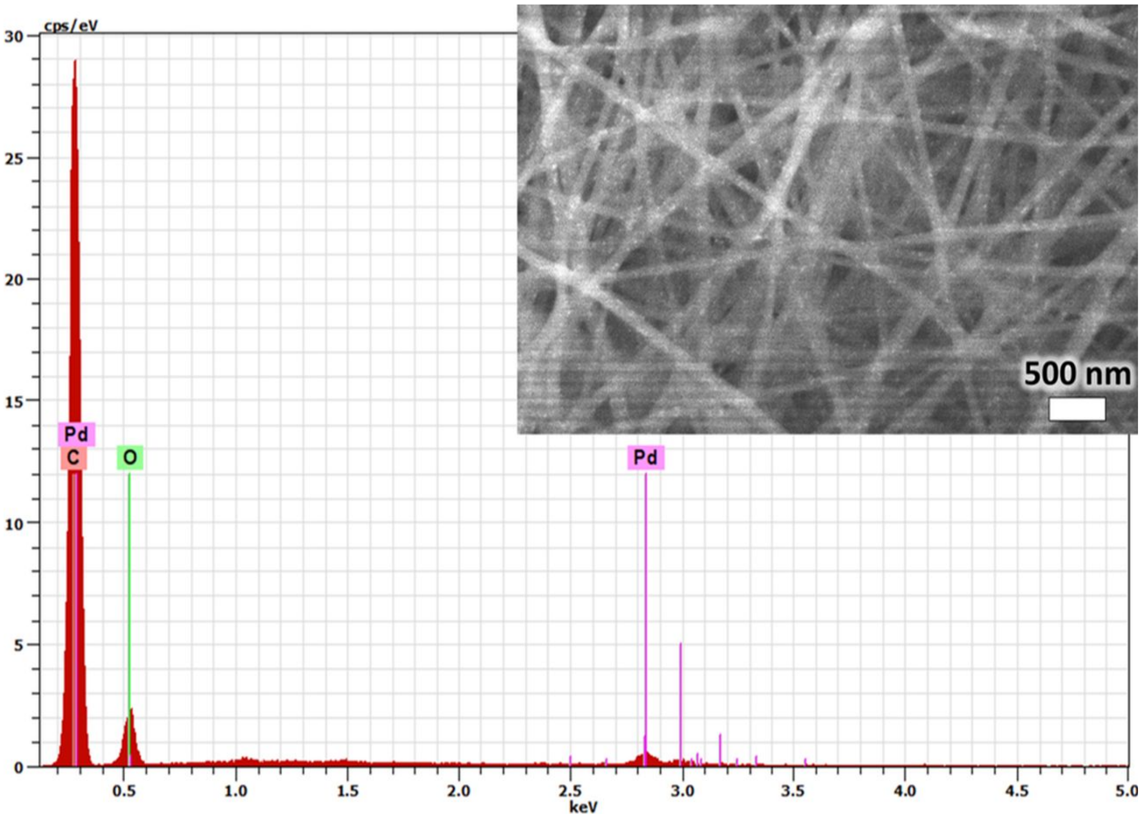

Figure. S2 EDX spectra of PVA-PdNPs fiber mat, inset showed the spot of EDX spectrum acquisition.

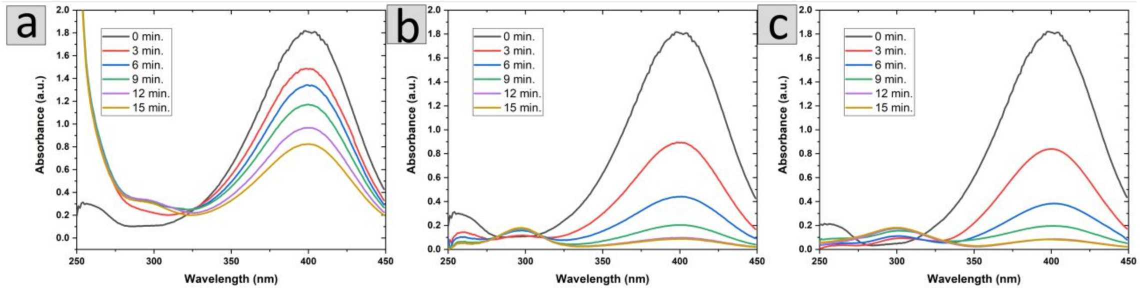

Figure. S3 Typical UV-Vis spectra recorded during 4-NP decomposition in the presence of nanofiber mat with: Pd NPs without stirring of the sample (A) and for 4th cycle (B) or PdNPs suspension (C).
